# *Panax quinquefolius L.* Saponins Protect Myocardial Ischemia Reperfusion No-Reflow Through Inhibiting the Activation of NLRP3 Inflammasome via TLR4/MyD88/NF-κB Signaling Pathway

**DOI:** 10.3389/fphar.2020.607813

**Published:** 2021-01-15

**Authors:** Ping Yu, Yuangeng Li, Wenwen Fu, Xin Li, Yanzhe Liu, Yaozhen Wang, Xiaofeng Yu, Huali Xu, Dayun Sui

**Affiliations:** Department of Pharmacology, School of Pharmaceutical Sciences, Jilin University, Changchun, China

**Keywords:** *panax quinquefolius* L. saponin, myocardial ischemia reperfusion no-reflow, inflammatory response, leukocytes, NLRP3 inflammasome

## Abstract

At present, many patients who undergo reperfusion immediately after percutaneous coronary intervention will undergo microvascular obstruction and reduction in myocardial blood flow. This phenomenon is called “no-reflow (NR),” and there is still no effective therapy for NR. Studies showed *Panax quinquefolius L.* saponins (PQS) have effect on MI/R injury, while the effect and mechanism of PQS on MI/R induced NR are not clear. In this study, we established a MI/R model to investigate whether PQS decrease NR phenomenon *via* suppression of inflammation. We found that PQS significantly alleviated the symptoms of NR by reducing ischemia, infarction, and NR area; improving cardiac function; preventing pathological morphology changes of myocardium; depressing leukocytes’ aggregation and adhesion; and suppressing the excessive inflammation. Further study demonstrated that PQS remarkably inhibited TLR4, MyD88, p-NF-κB, and NLRP3 inflammasome-associated protein, and these effects could be reversed by LPS. These results indicated that PQS may protect NR by inhibiting the activation of NLRP3 inflammasome *via* TLR4/MyD88/NF-κB signaling pathway in part, suggesting that PQS exist potential in preventing NR induced by MI/R.

## Introduction

Acute myocardial infarction (AMI) is the leading cause of death in the world; the prevalence and mortality rates are still on the rise. Thrombolysis and percutaneous coronary interventions (PCI) are still the most efficient treatments for coronary revascularization (Kalogeris et al., 2016). However, 10%–60% patients who undergo reperfusion immediately after the initial PCI will cause microvascular occlusion and the decrease of myocardial bloodstream, and this phenomenon is called “NR” (Kalogeris et al., 2012; Cinteza, 2019; Wang et al., 2020a). Previous research shows that reperfusion and NR are the main reasons for the deterioration of the cells exposed to the ischemic environment (Wang et al., 2020b). At the same time, it also leads to the poor prognosis of patients with acute myocardial infarction, such as cardiac arrhythmia, heart failure, and cardiogenic shock (Vrints, 2020; Wasmer et al., 2020). The factors associated with the establishment of NR include endothelial dysfunction, compression of capillaries by swollen myocytes, leukocyte adherence, microvascular ischemia, oxidative stress, and inflammation (Heusch, 2019; Wang et al., 2020b). Furthermore, accumulating evidence indicates inflammation is considered to be a promising direction, but its underlying molecular mechanisms are not clear; similarly, there are few effective drugs.

MI/R can cause severe acute inflammation (Liu et al., 2019; Lu et al., 2019; Yang et al., 2020). Specifically, during MI/R, with the reflow of oxygen and blood, the heart produces a large number of stress reactions. Although most of these reactions are beneficial, due to excessive protection and defense, the activation of the immune system and the production of sterile inflammatory reactions may lead to serious consequences, such as NR (Li et al., 2016; Ren et al., 2016; Li et al., 2019). However, this sterile inflammation is similar to the response caused by pathogen invasion, and the production of cytokines, chemokines, and other pro-inflammatory stimuli causes a number of neutrophils and other leukocytes to be recruited to the inflamed heart (Mazzoni et al., 1995; Dai et al., 2020; Liu et al., 2020). Leukocytes become less deformed in ischemic environment; at the same time, by the influence of adhesion molecules such as ICAM-1 and CD62L, a large number of leukocytes aggregate and tightly adhere to narrow capillaries (Huang et al., 2018). “Gigantic” leukocytes cannot pass through narrow capillaries without powerful bloodstream, which may cause microvascular blockage and lead to the occurrence of NR phenomenon. Recent evidence suggests that the sterile inflammatory response triggered by tissue damage is mediated by multiple protein complexes called NLRP3 inflammasome (Zhou et al., 2018; Zhao et al., 2020). The NLRP3 inflammasome contains NLRP3, ASC, and procaspase-1. In this complex, NLRP3 initiates the formation of an inflammasome by interacting with ASC that recruits and cleaves procaspase-1 to caspase-1. Caspase-1 is an IL-1β– and IL-18–converting enzyme that can cleave pro-IL-1β and pro-IL-18 into their active forms, IL-1β and IL-18 (An et al., 2019; Zuurbier, 2019). Aseptic inflammation triggers an inflammatory response by detecting damage associated molecular pattern (DAMP) *via* extracellular and intracellular pattern recognition receptors (Seong and Matzinger, 2004). Inflammasome is the initial sensors of danger signals in MI/R injury. Studies have found that the activation of inflammasome in cardiomyocytes is closely related to the initial inflammatory response after MI/R (Wang et al., 2017; Toldo et al., 2019). NLRP3 inflammasome is formed by MI/R, and its subsequent activation leads to the production of IL-1β and IL-18, leading to inflammatory reactions such as inflammatory cell infiltration, adhesion, and aggregation in the heart. TLR4/MyD88/NF-κB is a classic inflammatory signaling pathway (Wang et al., 2020c; Gao et al., 2020). During MI/R, the heart released endogenous danger signals and activated related signaling pathways represented by TLR4/MyD88/NF-κB via DAMP, and the TLR4/NF-kb/NLRP3 signaling pathway plays an important role in MI/R injury (Zhang et al., 2017), but there is no evidence that the occurrence of NR phenomenon after reperfusion is related to it. Therefore, it is necessary to explore the molecular mechanism of inflammation in NR phenomenon.

*Panax quinquefolius L.* is an important medicinal plant belonging to the Araliaceae family. *Panax quinquefolius L.* saponins (PQS) are extracted from American *Panax ginseng*. Pharmacological studies have found that PQS have the effects of improving immunity, anti-fatigue, anti-inflammation, *etc*. (Tang et al., 2016; Kim et al., 2019; Xing et al., 2019). Previous studies have shown that PQS can reduce MI/R injury by inhibiting the generation of reactive oxygen species and reducing inflammation (Li et al., 2014). However, the mechanisms for the cardioprotective effect of PQS remain poorly defined. We hypothesized that the NR phenomenon was induced by MI/R operation, and the TLR4/MyD88/NF-κB/NLRP3 inflammasome pathway–mediated inflammatory response may be related to PQS-mediated cardiac protection mechanisms.

## Materials and Methods

### High-Performance Liquid Chromatography Analysis of *Panax quinquefolius L.* saponins

High-performance liquid chromatography (HPLC) analysis was conducted to identify the main chemical components of PQS. HPLC was performed on Agilent 1,260 Sepax (Agilent Technologies, California, United States) equipped with a Sepax Bio-C18 column (250 × 4.6 mm, 5 µm). The column temperature was 40°C, and the injection volume was 10 μl. The mobile phase eluted with ultrapure water (A) and acetonitrile (B) in gradient mode. The proportion of methanol was varied from 19% to 35% in 100 mins (0–45 min, 19%–19% B; 45–50 min, 19%–27% B; 50–60 min, 27%–31% B; 60–70 min, 31%–28% B; 70–85 min, 28%–35% B; 85–100 min, 35%–35% B) at a flow rate of 1.0 ml/min. Samples were detected with a UV detector at a wavelength of 203 nm.

### Drug and Reagents

PQS (purity: >98%) was provided by Professor Yanping Chen (Department of Natural Medicinal Chemistry, Jilin University, Changchun, China). 2, 3, 5-Triphenyltetrazolium chloride (TTC), Evans blue, Thioflavin-S, and lipopolysaccharide (LPS) were bought from Sigma (St. Louis, MO, United States). The BCA protein assay kit, radio immunoprecipitation assay (RIPA) lysis buffer, QuickBlock™ Blocking Buffer for Western blot, and BeyoECL Plus were supplied by Shanghai Beyotime Biotechnology (Shanghai, China). Horseradish peroxidase (HRP) detection system and diaminobenzidine (DAB) were from ZSGB-BIO (Beijing, China). Lactate dehydrogenase (LDH) assay kit, myeloperoxidase (MPO) assay kit, creatine-MB (CK-MB) ELISA kit, cardiac troponin I (cTnI) ELISA kit, interleukin-1β (IL-1β) ELISA kit, interleukin-18 (IL-18) ELISA kit, tumor necrosis factor-α (TNF-α) ELISA kit, and interferon-γ (IFN-γ) ELISA kit were bought from Nanjing Jiancheng Bioengineering Institute (Nanjing, China). TRIzol^®^ was bought from Thermo Fisher Scientific (Waltham, MA, United States). TransScript Green Two-Step qRT-PCR SuperMix was purchased from Beijing TransGen Biotech (Beijing, China). The anti-β-Actin monoclonal antibody, HRP-conjugated anti-rabbit IgG, as well as primers of interleukin 18 (IL-18) and interleukin 1β (IL-1β) were from Beijing Dingguo Changsheng Biotechnology (Beijing, China). ICAM-1 antibody, CD62L antibody, NF-κB p65 antibody, and p-NF-κB p65 antibody were from Abcam (Cambridge, United Kingdom.). TLR4 antibody, MyD88 antibody, NLRP3 antibody, ASC antibody, and Caspase-1 antibody were from Affinity Biosciences (Cincinnati, United States). Other reagents were obtained from local distributors in the highest available quality.

### Animals and Experimental Groups

Wistar rats weighing 210–230 g, both male and female, 6–8 weeks of age, were provided by the Experimental Animal Center of Jilin University (Changchun, China). All rats were housed in the Animal Center of Jilin University in a standard environment. The experiments were performed in accordance with the Guide for the Care and Use of Laboratory Animals of Jilin University, and experimental protocols were approved by the Ethics Committee of Jilin University.

**FIGURE 1:**
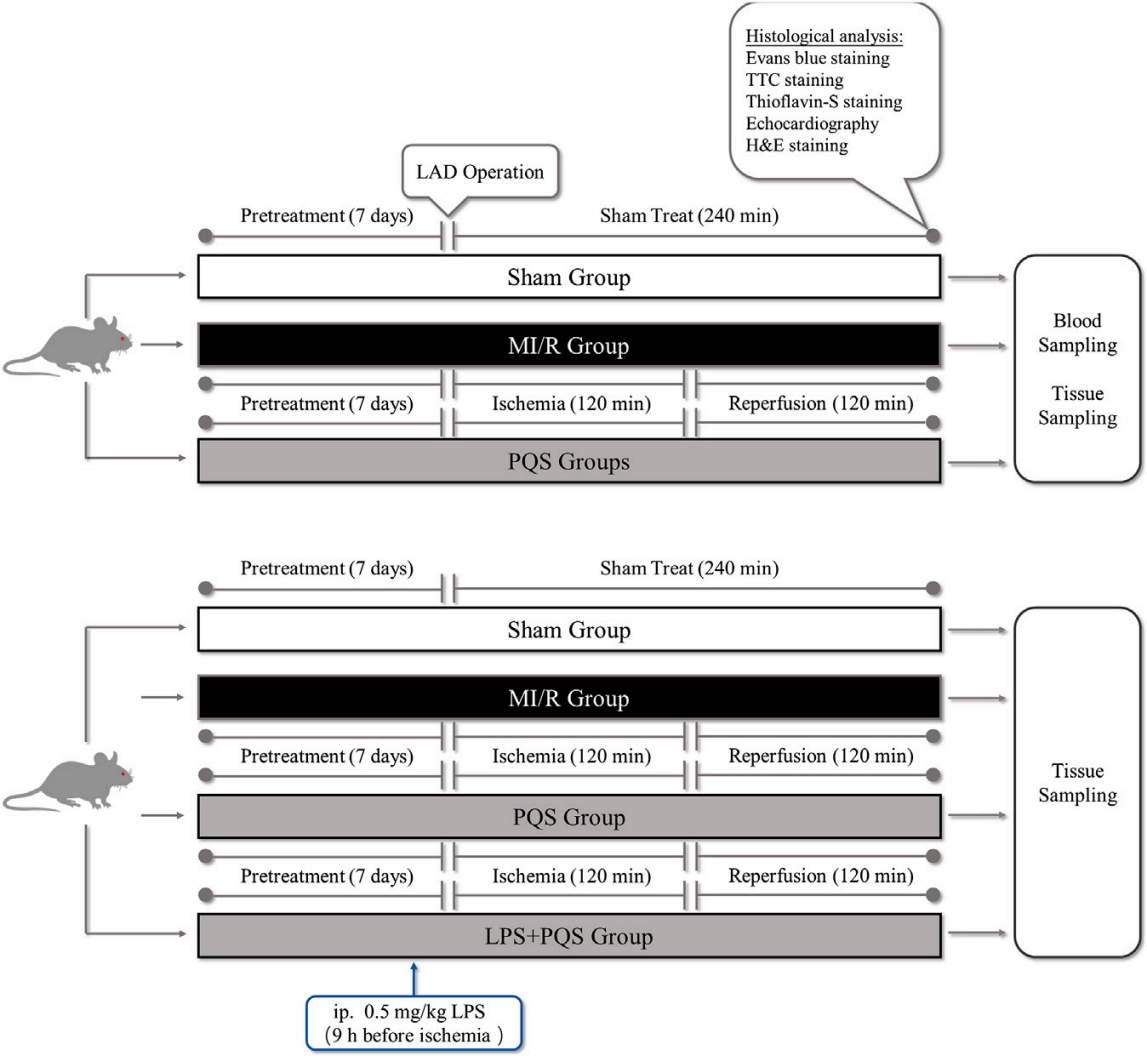
The experimental procedures of *in vivo* MI/R rat model. PQS groups indicate the MI/R group treated with 70, 140 mg/kg dose of PQS.

This study was divided into two parts as following (Figure 1): Part 1. To investigate the protective effect of PQS on NR phenomenon, rats were randomly divided into four groups: 1) Sham group, 2) MI/R group, 3) PQS (70 mg/kg) treatment group, and 4) PQS (140 mg/kg) treatment group. Part 2. To determine the mechanism on PQS, we performed LPS (TLR4 activator) experiment including the following groups: 1) Sham group, 2) MI/R group, 3) PQS (140 mg/kg) treatment group, and 4) PQS (140 mg/kg) + LPS treatment group. Rats were intragastrically administrated with PQS (once a day, 140 mg/kg) for 7 days. LPS (0.5 mg/kg dissolved in 0.9% saline solution) was administrated 9 h before ischemia by intraperitoneal injection (Vaez et al., 2016).

### Protocol of MI/R Induced No-Reflow Phenomenon

The MI/R animal model was established in accordance with the procedure in Figure 1 (Fu et al., 2018). Briefly, the rats were anaesthetized by intraperitoneal injection of 3% pentobarbital sodium for 30 mg/kg and received mechanical ventilation from an animal ventilator after endotracheal intubation. The chest was opened to expose the heart at the fourth intercostal space of the left subclavian midline, and then MI/R was induced by ligating the left anterior descending coronary artery (LAD) for 120 mins followed by reperfusion for 120 mins. The sham group underwent the same surgical procedures without LAD ligation.

### Echocardiography Measurement

To evaluate the cardiac function, various left ventricular functional indices, such as left ventricular internal dimension at diastole (LVIDd) and left ventricular internal dimension at systole (LVIDs), left ventricular fractional shortening (LVFS), and left ventricular ejection fraction (LVEF) were automatically measured and calculated by a 10 S scan head (Vivid I; GE Healthcare, Piscataway, NJ, United States). All parameters were gauged more than three consecutive cardiac cycles. Measurements were operated by a professional echocardiographer who was blinded to the experimental groups.

### Determination of Serums Myocardial Enzyme Level by Assay Kit

At the end of reperfusion, the blood was collected at 2,500 rpm/min for 10 mins to obtain the serum samples to measure the levels of CK-MB and cTnI using ELISA assay kits according to the manufacturer’s instructions. Serum LDH was determined using an LDH assay kit according to the manufacturer’s instructions.

### Myocardial Staining for Area at Risk, Area at Infarct, and Area at No-Reflow Evaluation

Measurements of the myocardial area at risk (AAR), area at infarct (AAI), and area at NR (AAN) were performed according to a previous report (Basalay et al., 2020; Chen et al., 2020). In brief, 4% thioflavin S 1 ml/kg was injected from the femoral vein, and 2% Evans blue 2 ml/kg was injected from the inferior vena cava. After cutting off the right ventricle and atrium, five slices of the left ventricle were transversely cut parallel to the atrioventricular sulcus with a thickness of about 2 mm. The ischemic area was observed: the non-blue stained area is the ischemic area, and the blue stained area is the nonischemic area. The no-reflow area was observed: the non-fluorescence area is the no-reflow area, and the fluorescent area is the reflow area. Then, we put the slices in 1% TTC solution, and fix them in 10% formalin for 20 mins at 37°C for 20 mins. The infarct area was observed: the infarct myocardial tissue is not colored, and the normal myocardial tissue is red.

We use Image-Pro Plus 6.0 to analyze the resulting pictures by selecting and measuring the area. The area of ischemia is expressed as proportions of ischemia myocardial to the whole left ventricular myocardial tissues (ischemia area/total left ventricular area) × percentage (AAR), the area of myocardial infarct is expressed as proportions of infarct myocardial to ischemia myocardial tissues (infarct area/ischemia area) × percentage (AAI), and the area of no-reflow is expressed as proportions of no-reflow myocardial to the whole left ventricular myocardial tissues (no-reflow area/total left ventricular area) × percentage (AAN).

### Histopathological Examination

At the end of reperfusion, the left ventricle of the hearts was collected and immediately fixed in 4% polyformaldehyde for 24 h after being washed with phosphate buffer saline. Then, embedded in the paraffin, the specimens were minced into 4 μm sections for hematoxylin-eosin (H&E) staining. Sections were observed under light microscope (Nikon, Japan). Each sample was observed at ×200 magnification.

### Peripheral Blood Leukocyte Counts

After reperfusion for 120 min, 20 μL blood were put into the EP tube, 3% glacial acetic acid solution was added into 180 μL of the solution, the EP tube was gently oscillated to make the blood, and the dilution was mixed well. And then, the leukocyte counts were counted under the microscope.

### Determination of Myocardial Tissue Inflammation Marker

At the end of reperfusion, the hearts were collected and immediately prepared into 10% tissue homogenates. The protein concentration was determined using the Bradford protein assay kit, the activity of MPO was determined by MPO assay kit, and the levels of IL-1β, IL-18, TNF-α, and IFN-γ were determined by ELISA assay kit according to the manufacturer’s instructions.

### Immunohistochemistry

For immunohistochemistry detection, after reperfusion, tissues from the heart region were collected and fixed with 4% paraformaldehyde for 24 h, and embedded in paraffin. Heart histology was assessed by immunohistochemistry with primary antibodies against ICAM-1 (1:200) or CD62L (1:200) under an upright Metallurgical Microscope (Nikon, Japan). At least three different sections from each specimen were examined.

### Quantitative Real-Time Polymerase Chain Reaction Assay

Total RNA was isolated from the heart of the rats from each group with the TRIzol Reagent, according to the standard protocol, mixed with chloroform by gently swirling, and centrifuged to obtain the upper aqueous phase. Isopropyl alcohol was added, the cells were centrifuged for sedimentary RNA, washed with 75% ethanol, centrifuged again, and then resuspended in diethyl pyrocarbonate (DEPC) water. The OD value ratio was detected to be 260 nm/280 nm. Subsequently, RNA was reverse-transcribed with oligo (dT) primers, and qPCR was conducted with gene-specific primers in the presence of SYBR Premix Ex Taq. qPCR was conducted for three independent experiments, using GAPDH as the housekeeping control. qPCR amplification was performed with 40–50 cycles (95°C, 5 s; 55°C, 15 s; 72°C, 10 s), and the oligonucleotide primer sets were as follows: The sequences of the primers are shown in Table 1.

**TABLE 1:** Primer sequences required for qRT-PCR.

Gene	Forward primer (5′–3′)	Reverse primer (5′–3′)
IL-1β	GAC​TTC​ACC​ATG​GAA​CCC​GT	GGA​GAC​TGC​CCA​TTC​TCG​AC
IL-18	ACC​GAA​CAG​CCA​ACG​AAT​CC	CAG​ATA​GGG​TCA​CAG​CCA​GTC
GAPDH	AGT​GCC​AGC​CTC​GTC​TCA​TA	TCC​CGT​TGA​TGA​CCA​GCT​TC

### Western Blot Analysis

After reperfusion, tissues were collected from the apical heart region, the lysate was inserted into 100 mg broken tissues, and then tissue proteins were extracted. The protein concentration was determined using the BCA protein assay kit. The protein was separated by SDS-PAGE and then transferred to the PVDF membrane. After blockaged with 5% nonfat milk, the PVDF membrane was incubated by TLR4 (1:1,000), MyD88 (1:1,000), NF-κB p65 (1:1,000), p-NF-κB p65 (1:1,000), NLRP3 (1:1,000), ASC (1:1,000), caspase-1 (1:1,000), and β-actin (1:1,000) overnight at 4°C and then incubated with the second antibody (1:5,000) for 1 h. The bands were visualized using ECL chemiluminescence. ImageJ software (version 1.5.0.26; National Institutes of Health, Bethesda, MD, United States) was used for analysis.

### Statistical Analysis

All values were expressed as the means ± standard deviation. Differences among experimental groups were analyzed by one-way ANOVA followed by a Bonferroni post hoc test. *p <* 0.05 were considered to be statistically significant. Statistical tests were performed using GraphPad Prism version 8.0 (GraphPad Software, United States).

## Results

### High-Performance Liquid Chromatography Analysis of *Panax quinquefolius L.* saponins

The main components of PQS were analyzed by HPLC. Among the components discovered in PQS, a total of nine components were identified according to their retention times, and their contents were also counted (Table 2).

**TABLE 2:** Retention times and contents of nine components in PQS.

Constituents	Retention times (min)	Contents (%)
Rg1	30.65	1.28
Re	32.38	2.75
Rf	60.36	0.20
Rg2	65.60	0.93
Rb1	69.88	2.66
Rc	77.39	1.18
Rb2	82.58	3.49
Rb3	84.08	10.59
Rd	89.05	10.36

### *Panax quinquefolius L.* saponins Improved Cardiac Function After MI/R

**FIGURE 2:**
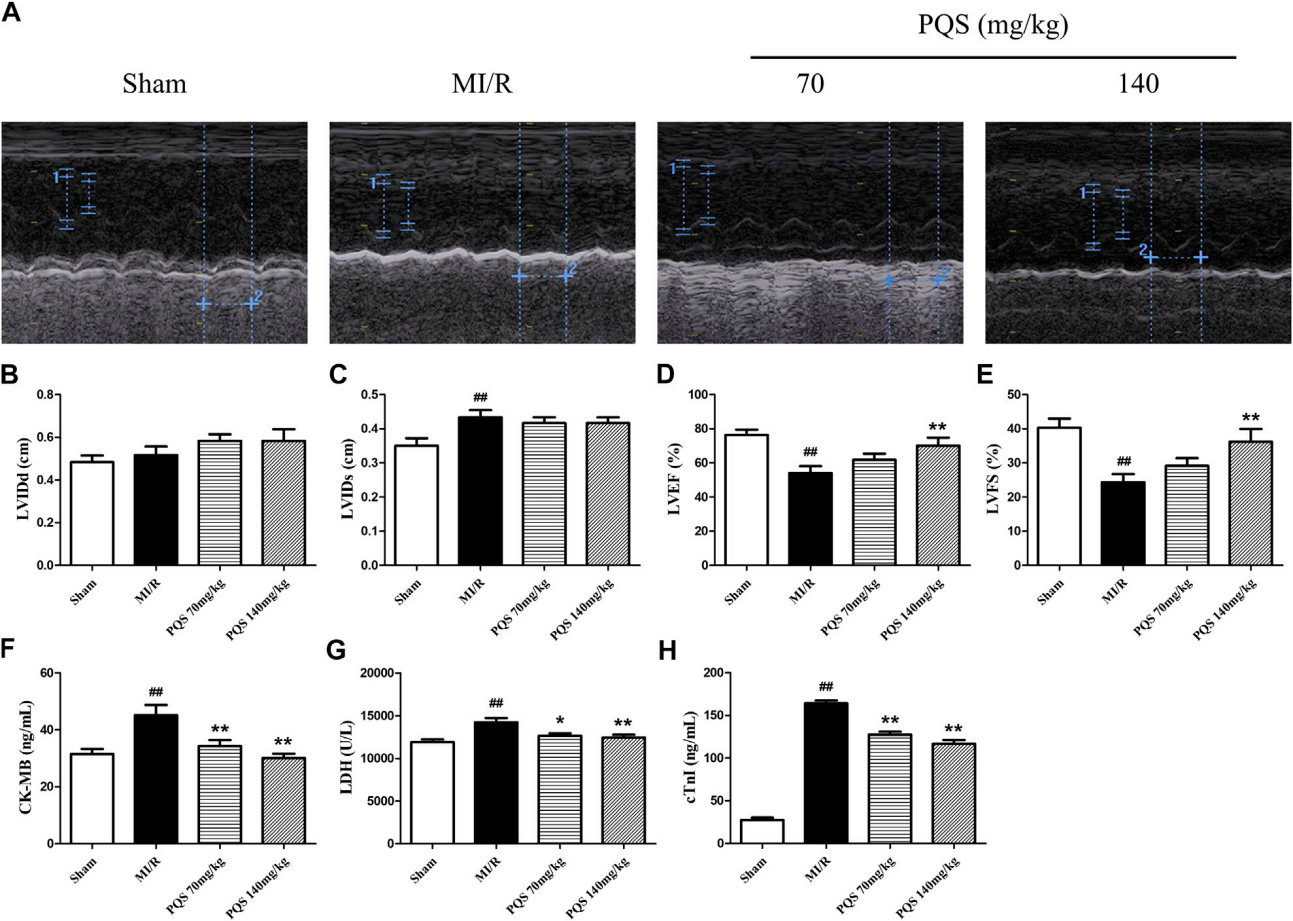
Effects of PQS on MI/R injury. **(A)** The representative M-mode photographs of cardiac function, **(B)** left ventricular internal dimension at the diastole (LVIDd), **(C)** left ventricular internal dimension at the systole (LVIDs), **(D)** left ventricular ejection fraction (LVEF), and **(E)** left ventricular fractional shortening (LVFS) in rat hearts were measured using Vivid I data acquisition device (n = 6 in each group). The levels of **(F)** CK-MB and **(G)** LDH and **(H)** cTnI in serum were determined by commercial kits (n = 10 in each group). Data presented are the means ± SD. ^#^ *p <* 0.05, ^##^ *p <* 0.01 compared with Sham; *p < 0.05, **p < 0.01 compared with MI/R.

As was shown in Figure 2A–E, the function of cardiac pump was impaired, and the left ventricular systolic function exhibited remarkable deterioration in MI/R rats. Compared with Sham group, LVEF and LVFS of MI/R group were significantly decreased, while LVIDs were obviously increased. PQS (140 mg/kg) could significantly increase LVEF and LVFS, and PQS (70 and 140 mg/kg) could reduce LVIDs to varying degrees.

### *Panax quinquefolius L.* saponins Repressed Serum Creatine-MB, Lactate Dehydrogenase, and cardiac troponin I Levels After MI/R

CK-MB, LDH, and cTnI were extremely important for evaluating the injury. As was shown in Figure 2F–H, CK-MB, LDH, and cTnI levels in the serum of MI/R group were significantly increased, indicating that the myocardial cell injury was severe in MI/R rat. Compared with the MI/R group, PQS (70 and 140 mg/kg) could reduce the levels of CK-MB, LDH, and cTnI in serum after MI/R.

### *Panax quinquefolius L.* saponins Decreased Cardiac Area At Risk, Area At Infarct, and Area At No-Reflow After MI/R

**FIGURE 3:**
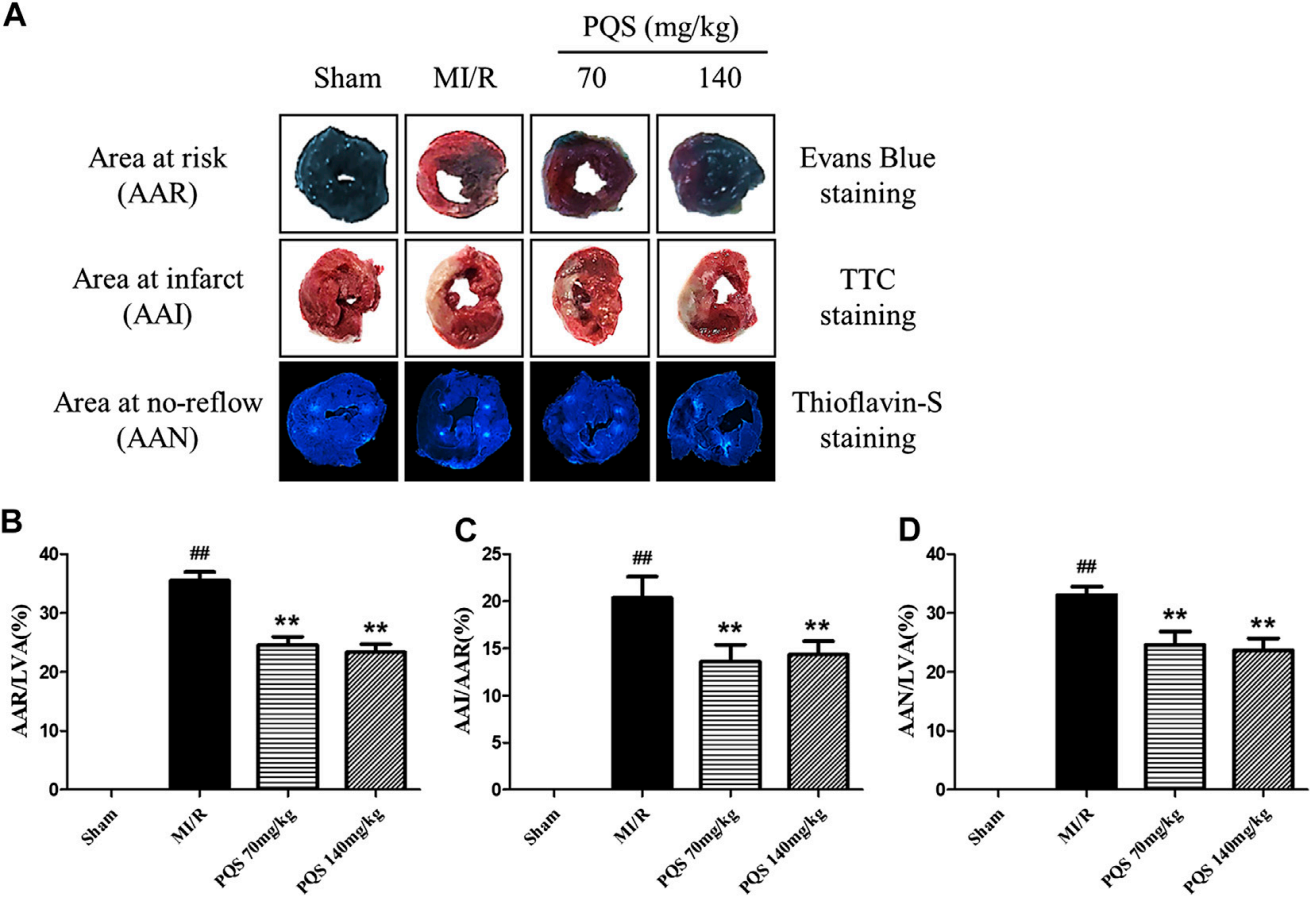
Effects of PQS on AAR/LVA, AAI/AAR, and AAN/LVA. **(A)** The representative heart morphological photographs of PQS on AAR stained by Evans blue, AAI stained by TTC, and AAN stained by thioflavin-S in rats; **(B)** AAR/LVA; **(C)** AAI/AAR; and **(D)** AAN/LVA. Data presented are the means ± SD. ##*p <* 0.01 compared with Sham; **p < 0.01 compared with MI/R.

As was shown in Figure 3A–D that all of the rats in Sham groups were found little ischemia, infarction, and NR area, therefore, the influence of operative technique on experimental results was excluded. AAR/LVA, AAI/AAR, and AAN/LVA in MI/R group were significantly increased. However, compared with the MI/R group, PQS (70 and 140 mg/kg) could significantly reduce rat myocardium AAR/LVA, AAI/AAR, and AAN/LVA. According to NR area results, the NR phenomenon was found in rats which underwent MI/R operation. Therefore, the NR phenomenon was successfully induced by MI/R operation.

### *Panax quinquefolius L.* saponins Ameliorated Myocardial Pathological Morphology

**FIGURE 4:**
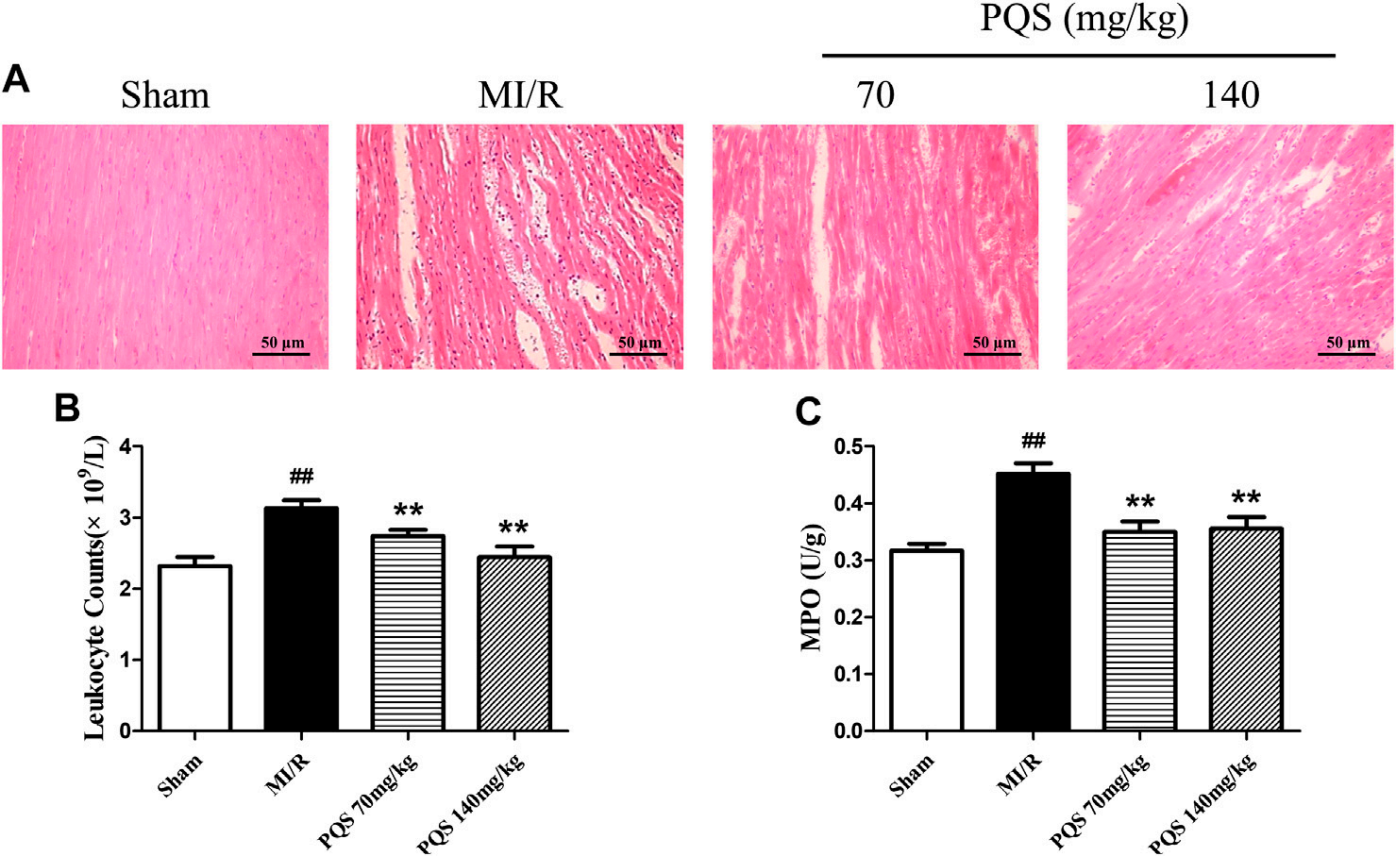
Effects of PQS on the histopathological changes, myocardial leukocyte counting, and MPO activity. **(A)** The representative light microscopic appearance of rat myocardial histopathological morphology (HE staining; original magnification ×200), **(B)** effect of PQS on myocardial leukocytes counting, and **(C)** effect of PQS on MPO activity. Data presented are the means ± SD. ##*p <* 0.01 compared with Sham; *p < 0.05, **p < 0.01 compared with MI/R.

As the H&E staining results showed in Figure 4A, the tissue in injury area was damaged severely, and myocardial fiber was fractured in MI/R group. However, PQS could ameliorate the myocardial histopathological changes obviously, which manifested as alleviative myocardial inflammatory infiltration and recoverable tissue structure.

### *Panax quinquefolius L.* saponins Reduced Peripheral Leukocytes and Myocardial myeloperoxidase Level

As was shown in Figure 4B,C, leukocyte counts and the level of MPO increased significantly in the MI/R group. Compared with the MI/R group, PQS (70 and 140 mg/kg) could significantly reduce rat leukocytes and the level of MPO. PQS could relieve both the number and activity of leukocytes.

### *Panax quinquefolius L.* saponins Down-Regulated Expression of Adhesive Molecules

**FIGURE 5:**
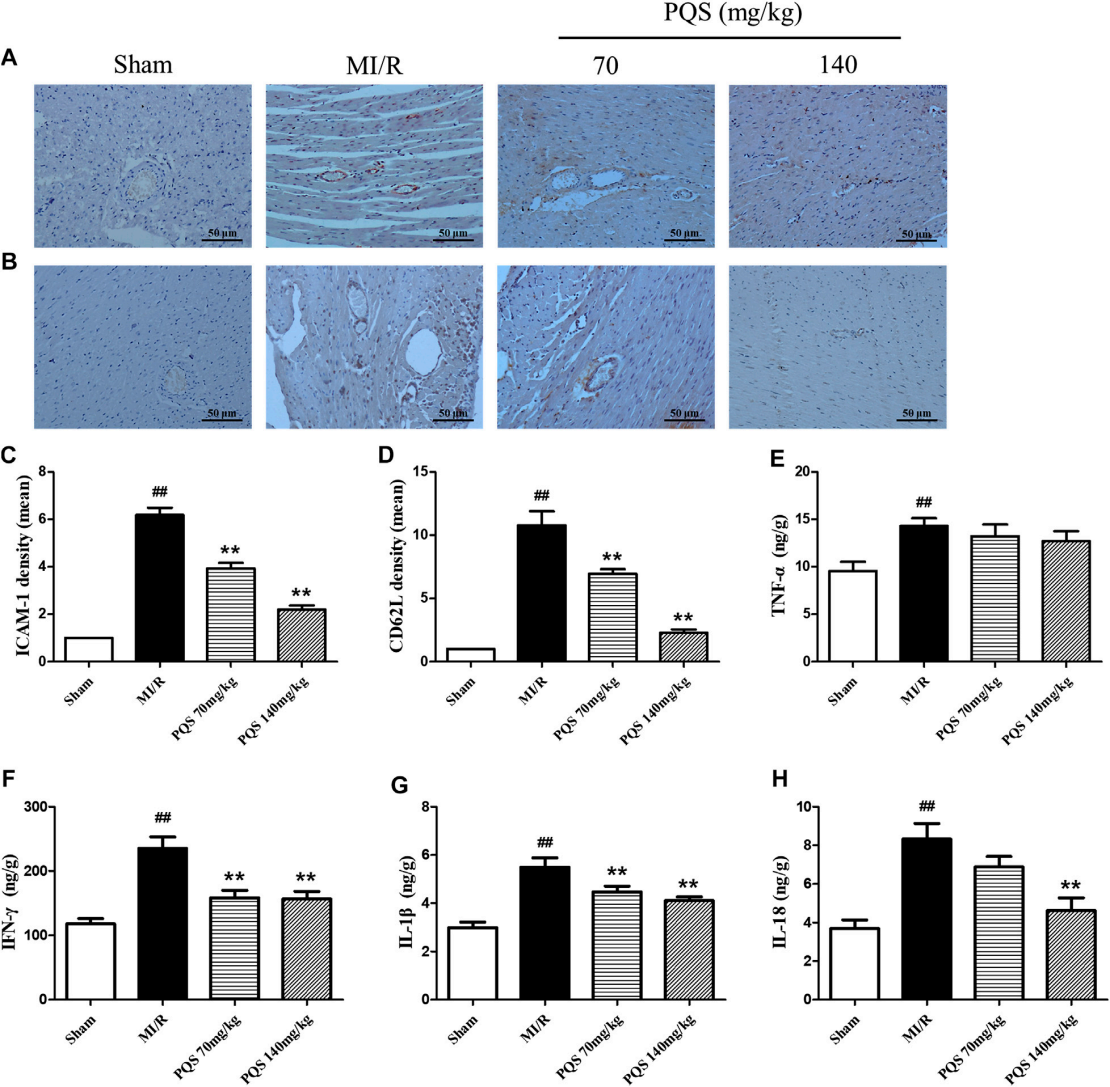
Effects of PQS on ICAM-1, CD62L, and inflammatory cytokine expressions after MI/R in myocardial tissue. The tissue was observed using a microscope at a magnification ×200. **(A)** Representative ICAM-1, **(B)** representative CD62L, **(C)** the expression of ICAM-1, **(D)** the expression of CD62L, and the levels of **(E)** TNF-α, **(F)** IFN-γ, **(G)** IL-1β, and **(H)** IL-18 in myocardial tissue were determined by commercial kits. Data presented are the means ± SD. ##*p <* 0.01 compared with Sham; *p < 0.05, **p < 0.01 compared with MI/R.

ICAM-1 is an important factor for the adhesion of leukocytes to vascular endothelial cells. As was shown in Figure 5A–D, the expressions of ICAM-1 and CD62L were significantly increased in the MI/R group. PQS (70, 140 mg/kg) could significantly decrease the expressions of ICAM-1 and CD62L compared to the MI/R group.

### *Panax quinquefolius L.* saponins Down-Regulated Inflammatory Response

Inflammatory factors played an important role both in the production of leukocytes and the subsequent inflammatory response caused by leukocytes. As was shown in Figure 5E–H, the levels of TNF-α, IFN-γ, IL-1β and IL-18 were significantly increased in the heart tissue of MI/R group, indicating that inflammatory response was serious in NR phenomenon. Compared with MI/R group, PQS (70 and 140 mg/kg) could reduce the levels of IL-1β and IFN-γ, and only PQS (140 mg/kg) reduced the level of IL-18, while PQS could not influence the level of TNF-α.

### *Panax quinquefolius L.* saponins Protected No-Reflow Phenomenon From Inhibiting NLRP3 Inflammasome Activation and Assembly

**FIGURE 6:**
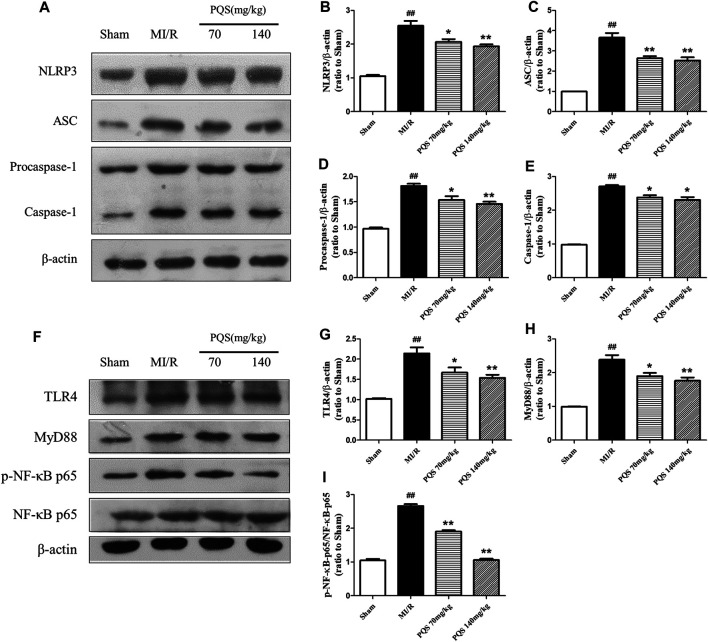
Effects of PQS on NLRP3, ASC, procaspase-1, and caspase-1 expressions. **(A)** Representative examples of Western blot analysis demonstrating the expression levels of NLRP3, ASC, procaspase-1, and caspase-1; quantification of the expression levels of **(B)** NLRP3, **(C)** ASC, and **(D)** procaspase-1 and **(E)** caspase-1; **(F)** representative examples of Western blot analysis demonstrating the expression levels of TLR4, MyD88, p-NF-κB, and NF-κB p65; quantification of the expression levels of **(G)** TLR4, **(H)** MyD88, **(I)** p-NF-κB, and NF-κB p65. Data presented are the means ± SD. ##*p <* 0.01 compared with Sham; *p < 0.05, **p < 0.01 compared with MI/R.

According to the above results, PQS significantly inhibited the production of IL-1β and IL-18; therefore, we focused our attention on NLRP3 inflammasome in which the end products were IL-1β and IL-18. As was shown in Figure 6A–E, the expressions of NLRP3, ASC, procaspase-1, and caspase-1 were diminished markedly in PQS (70 and 140 mg/kg) groups compared to the MI/R group. And then, we determined upstream proteins of NLRP3 inflammasome. As was shown in Figure 6F–I, the expressions of TLR4, MyD88, and p-NF-κB p65 were decreased in PQS (70 and 140 mg/kg) groups compared to the MI/R group. These results indicated that PQS significantly inhibited NLRP3 inflammasome activation and assembly, which may be related to TLR4/MyD88/NF-κB signaling pathway.

### *Panax quinquefolius L.* saponins Protected No-Reflow Hearts From Inhibiting NLRP3 Inflammasome *via* TLR4/MyD88/NF-κB Signaling Pathway

**FIGURE 7:**
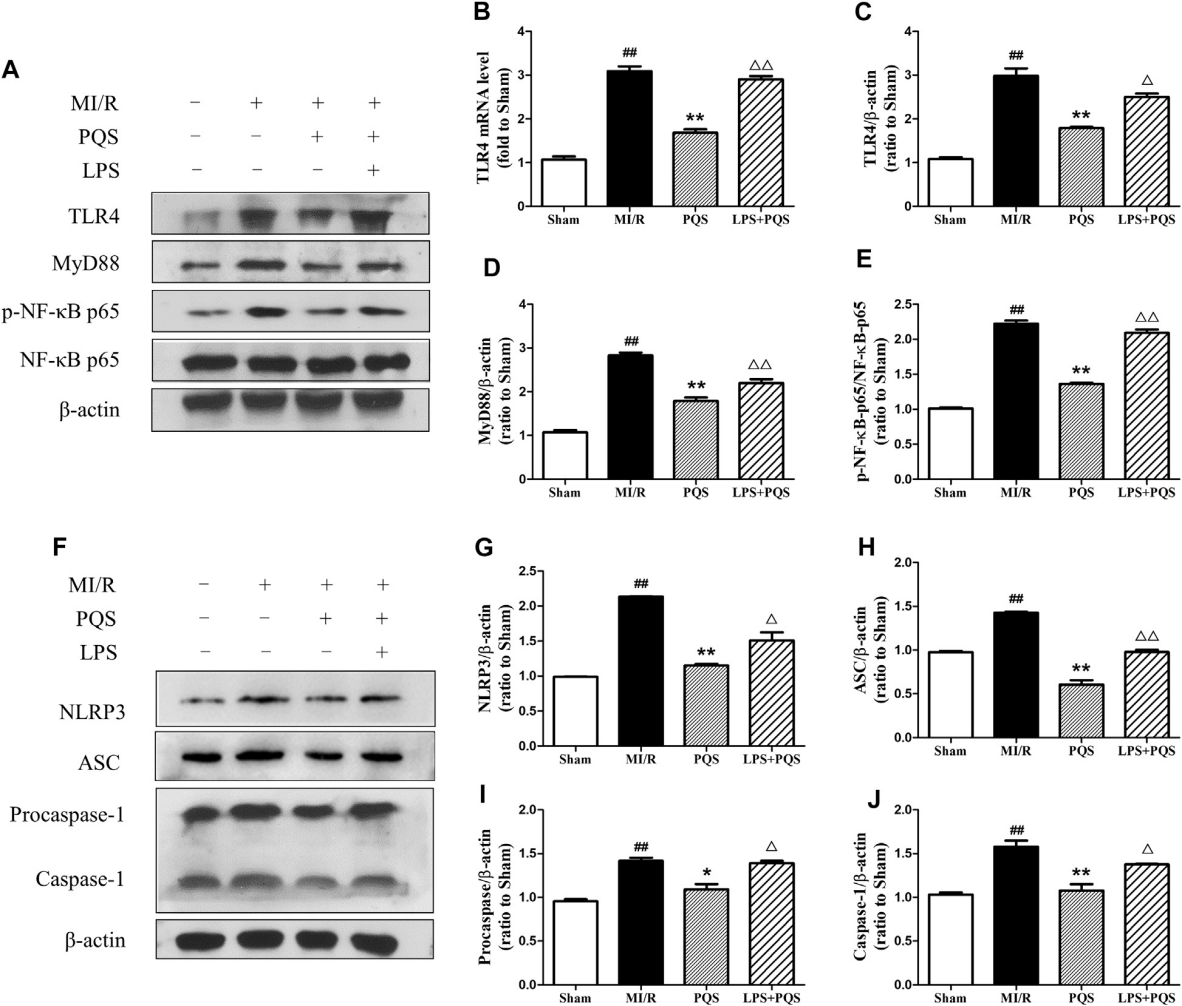
PQS inhibited the activation of NLRP3 inflammasome *via* TLR4/MyD88/NF-κB signaling pathway in the NR phenomenon. **(A)** Representative examples of Western blot analysis demonstrating the expression levels of TLR4, MyD88, p-NF-κB p65, and NF-κB p65; **(B)** the mRNA level of TLR4; quantification of the expression levels of **(C)** TLR4, **(D)** MyD88, **(E)** p-NF-κB p65, and NF-κB p65; **(F)** representative examples of Western blot analysis demonstrating the expression levels of NLRP3, ASC, procaspase-1, and caspase-1; quantification of the expression levels of **(G)** NLRP3, **(H)** ASC, **(I)** procaspase-1, and **(J)** caspase-1. Data presented are the means ± SD. ##*p <* 0.01 compared with Sham; *p < 0.05, **p < 0.01 compared with MI/R; ∆∆*p <* 0.01 compared with PQS.

In order to explore the mechanism of PQS influenced NLRP3 inflammasome, we used LPS to active TLR4/MyD88/NF-κB signaling pathway. As was shown in Figure 7A–J, the expressions of TLR4, MyD88, and p-NF-κB p65 were reduced obviously in PQS (140 mg/kg) groups compared to the MI/R group. However, after injection of LPS, all the expressions of these proteins were raised to the level of the MI/R group. At the same time, LPS could also increase the expression of NLRP3, ASC, procaspase-1, and caspase-1. As was expected, the changes of mRNA level of TLR4 are similar to the expression of its protein. These results indicated that PQS may protect NR from inhibiting NLRP3 inflammasome *via* TLR4/MyD88/NF-κB signaling pathway.

### *Panax quinquefolius L.* saponins Reversed No-Reflow Phenomenon and Inflammation Aggravated by Lipopolysaccharide

**FIGURE 8:**
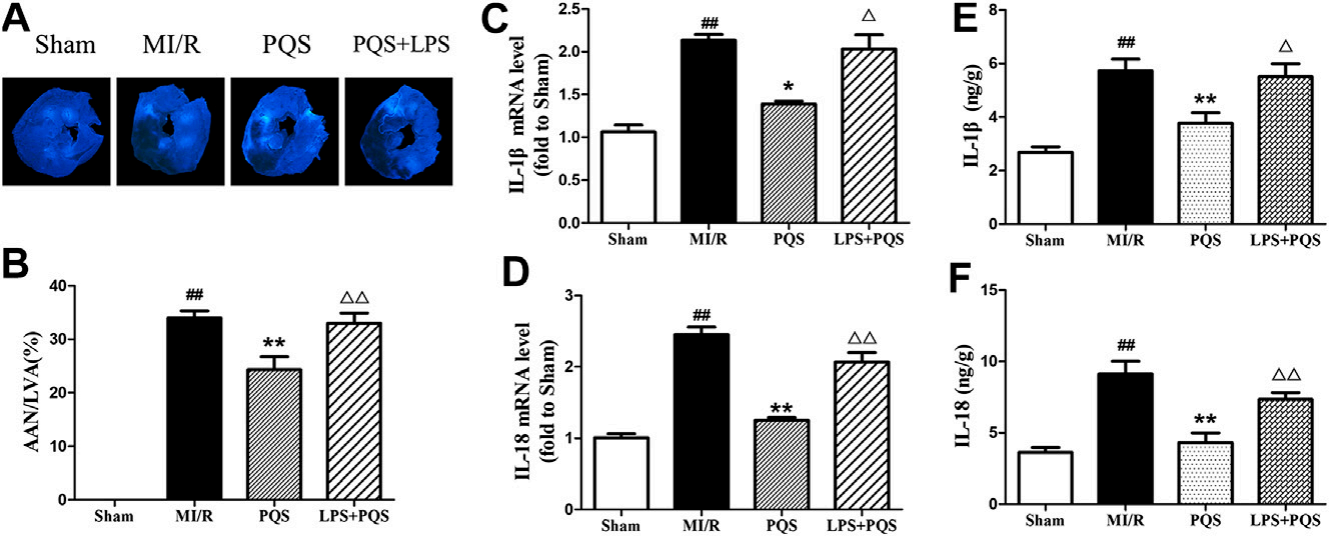
LPS reversed the effects of PQS on the NR phenomenon, inflammatory cytokines mRNA levels and contents. **(A)** The representative NR photographs; **(B)** AAN/LVA; the mRNA levels of **(C)** IL-1β and **(D)** IL-18 were determined by commercial kits. The contents of **(E)** IL-1β and **(F)** IL-18 were determined by ELISA kits. Data presented are the means ± SD. ##*p <* 0.01 compared with Sham; *p < 0.05, **p < 0.01 compared with MI/R; ∆*p <* 0.05, ∆∆*p <* 0.01 compared with PQS.

As was shown in Figure 8A–F, compared to the PQS group, LPS significantly increased the AAN, mRNA levels, and contents of IL-1β and IL-18. These results indicated that PQS could reverse the NR phenomenon and inflammation which were aggravated by LPS.

## Discussion

**FIGURE 9:**
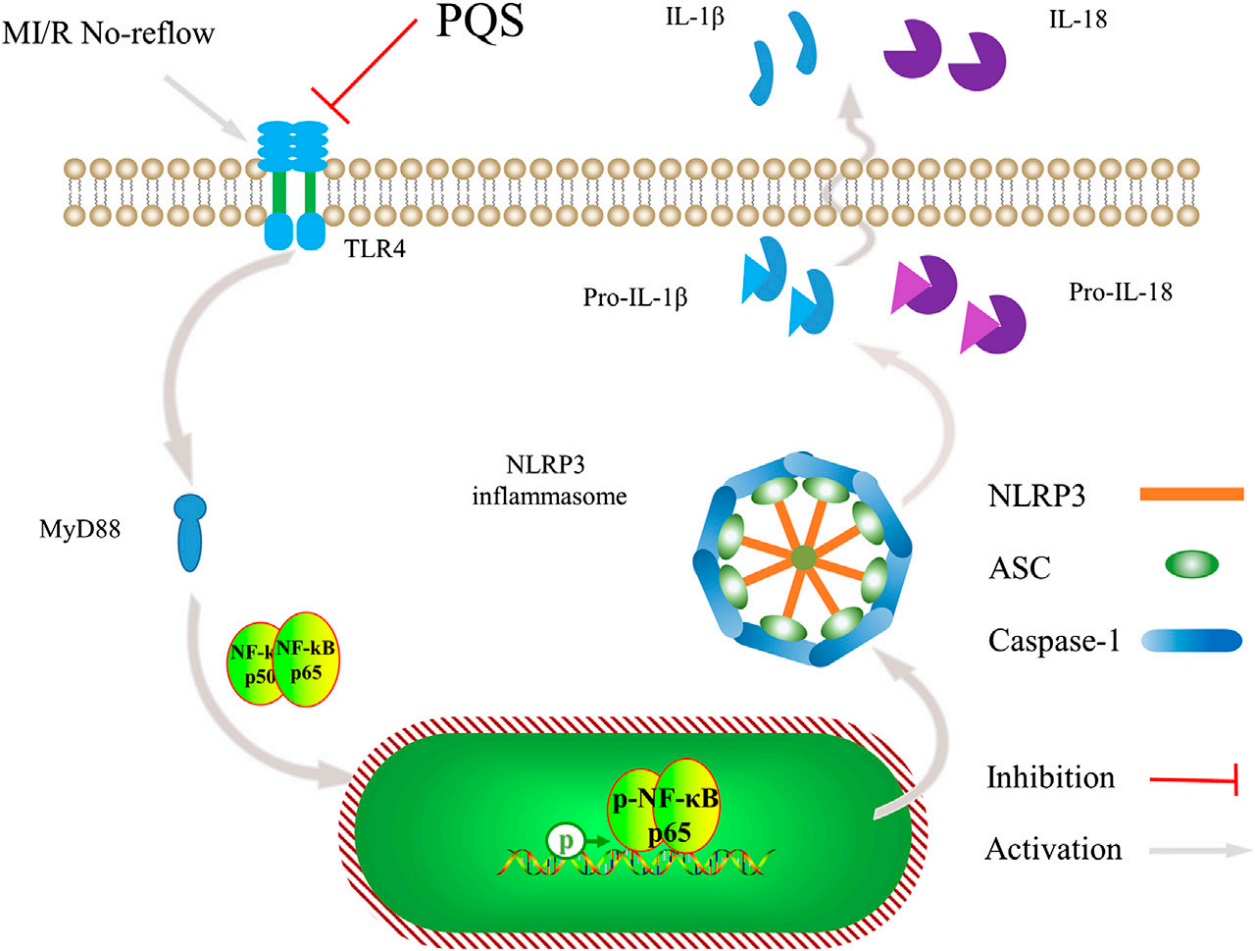
Schematic diagram describing the mechanism in the inhibitory effect of PQS on NR inflammatory injury.

In the current study, we have noted that administration of PQS can ameliorate MI/R injury and exhibit optimal cardioprotective effect. As far as we know, our study provides evidence for the first time that PQS show beneficial effect on MI/R NR phenomenon. This study clearly demonstrates that PQS can reduce NR area in MI/R rats and protect the damaged heart by improving cardiac function. In addition, our results indicate that inhibiting the activation of NLRP3 inflammasome through TLR4/MyD88/NF-κB signaling pathway is involved in this protective process (Figure 9).

Myocardial infarction is a common acute ischemic heart disease. Usually, thrombolytic therapy and PCI are the main methods for clinical reconstruction of blood supply (Botker, 2019). It is extremely difficult to rebuild the natural myocardial infarction in experimental animals. Therefore, most animal models of the coronary artery LAD are ligated by surgery, and then the ligation is released to simulate the reperfusion and the NR phenomenon of PCI patients (Heusch, 2017). However, the occurrence time of NR after reperfusion in this model is still unclear. Studies have shown that the lasting time of both ischemia and reperfusion are extremely important for the occurrence of NR. It was found in the previous studies that the NR phenomenon occurred at different time of ischemia (0.5–4 h) and reperfusion (1–72 h) (O’Farrell et al., 2017). In our study, we find that microvascular injury and NR phenomenon will occur obviously when ischemia lasts 120 mins and reperfusion lasts 120 mins. In such conditions, we established MI/R model to explore the possibility of PQS to attenuate the NR phenomenon. Our results manifested that PQS were able to decrease the area at NR determined by thioflavin-S staining after MI/R. Furthermore, ischemic and infarct area were reduced with cardiac dysfunction prevented, and myocardial enzymes and cTnI improved.

Inflammation plays an important role in many cardiovascular diseases. However, the process of MI/R may lead to the inflammatory response which may aggravate the MI/R injury in turn, like NR phenomenon. Leukocytes are involved in multiple noninfectious inflammatory processes including the response to MI/R, which serve as a key effector in the immune system (Jerome et al., 1994a; Cuijpers et al., 2020). In our study, according to histopathological determination and the counting of leukocytes, pretreatment with PQS could significantly increase leukocytes counts and attenuate leukocytes infiltration. MPO is considered to be the most effective indicator of lymphocytes. Similarly, the activity of MPO was decreased after pretreatment with PQS. The levels of inflammatory factors can directly reflect the intensity of inflammatory response. In this study, we found the levels of IL-1β and IL-18 in MI/R rats were significantly reduced by the pretreatment with PQS. These findings provide a potential link between PQS’ MI/R NR protective effect and inflammation inhibitory property.

So far, observed results indicate that NR phenomenon is not caused by blockage of platelets or fibrin thrombi (Kalogeris et al., 2016). The main reason may be a large number of leukocytes exposed to the acidic environment of ischemic tissues, and the cell structure becomes stiff (Mazzoni et al., 1995). At the same time, by the recruitment of inflammatory cytokines and chemokines, a large number of leukocytes accumulate to the damaged part, further aggravating inflammation, which leads to the aggravation of microvascular damage, thereby intensifying no-reflow (Kalogeris et al., 2016). Therefore, during ischemia, the blood flow through the capillaries is reduced, and with the effect of adhesion factors, such as ICAM-1 and CD62L, these leukocytes are more likely to sink into the capillaries, and eventually lead to NR (Jerome et al., 1994b). In our study, the expressions of ICAM-1 and CD62L in cardiac tissue were significantly reduced by PQS, and ICAM-1 was mainly expressed on the inner wall of blood vessels as previous studies reported (Jerome et al., 1994b). Furthermore, we found that PQS could decrease the adhesion and aggregation of leukocytes in microvessels, which may promote the aggravation of inflammation and microvascular damage. These results contributed to the protective effect of PQS in microvascular occlusion and NR phenomenon.

NLRP3 inflammasome is a multi-protein complex located in the cytoplasm, which is composed of NOD-like receptor protein 3 (NLRP3), apoptosis-related dot-like protein (ASC), and cysteine aspartase-1 precursor (Procaspase-1). Evidence was given that assembling and activation of NLRP3 inflammasome occurred at the most stages of MI/R process and then sequentially modulated the occurrence of downstream inflammatory factors, IL-1β and IL-18 (Guo et al., 2016). In the present study, our results showed that, with the occurrence of NR phenomenon, NLRP3 inflammasome was obviously activated, and pretreatment of PQS significantly inhibited the expression of NLRP3, ASC, and caspase-1. These findings initially prove that PQS’ MI/R NR protective effect is related to inhibiting the activation of NLRP3 inflammasome.

As is known, NLRP3 inflammasome locates in the cytoplasm, and we presume that there may exist a signaling pathway that transmits signals from the outside to the inside. TLR4/MyD88/NF-κB is known as a classical signaling pathway whose activation is thought to be responsible for the massive inflammation in MI/R injury and considered to be a valuable and promising therapeutic target against MI/R injury (Yang et al., 2016). Interestingly, we found that PQS significantly down-regulated the expression of TLR4, MyD88, and p-NF-κB compared with MI/R group. According to the fact, we concluded that TLR4/MyD88/NF-κB signaling pathway plays an important role in PQS’ MI/R NR protective effect. Moreover, carbohydrates of bacterial endotoxin (LPS) interacting with the host Toll-like receptor 4/myeloid differentiation factor 2 (TLR4/MD-2) complex which comprise the TLR4/MD-2/LPS complex is the first step in the inflammatory process (Cochet and Peri, 2017). As Haleh Vaez did, we injected LPS to active TLR4 to verify our hypothesis (Vaez et al., 2016). As we expected, the inhibiting effect of NLRP3 inflammasome by PQS was partly reversed. Consequently, it was evident that PQS may reduce NR phenomenon from MI/R through inhibiting NLRP3 inflammasome partly involved in TLR4/MyD88/NF-κB signaling pathway.

## Data Availability

The datasets presented in this study can be found in online repositories. The names of the repository/repositories and accession number(s) can be found in the article/Supplementary Material.
